# Some Endpoint Results for **β**-Generalized Weak Contractive Multifunctions

**DOI:** 10.1155/2013/948472

**Published:** 2013-11-04

**Authors:** H. Alikhani, D. Gopal, M. A. Miandaragh, Sh. Rezapour, N. Shahzad

**Affiliations:** ^1^Department of Mathematics, Azarbaijan University of Shahid Madani, Azarshahr, Tabriz, Iran; ^2^Department of Applied Mathematics and Humanities, S. V. National Institute of Technology, Surat, Gujarat 395007, India; ^3^Department of Mathematics, King Abdulaziz University, P.O. Box 80203, Jeddah 21859, Saudi Arabia

## Abstract

We introduce **β**-generalized weak contractive multifunctions and give some results about endpoints of the multifunctions. Also, we give some results about role of a point in the existence of endpoints.

## 1. Introduction

Let (*X*, *d*) be a metric space, *CB*(*X*) the collection of all nonempty bounded and closed subsets of *X*, and *H* the Hausdorff metric with respect to *d*; that is, *H*(*A*, *B*) = max⁡{sup_*x*∈*A*_ *d*(*x*, *B*), sup_*y*∈*B*_ *d*(*y*, *A*)} for all *A*, *B*∈*CB*(*X*), where *d*(*x*, *B*) = inf⁡_*y*∈*B*_ *d*(*x*, *y*). Let *T* : *X* → 2^*X*^ be a multifunction. An element *x* ∈ *X* is said to be a fixed point of *T* whenever *x* ∈ *Tx*. Also, an element *x* ∈ *X* is said to be an endpoint of *T* whenever *Tx* = {*x*} [1]. We say that *T* has the approximate endpoint property whenever inf⁡_*x*∈*X*_sup⁡_*y*∈*Tx*_ *d*(*x*, *y*) = 0 [1]. Let *f* : *X* → *X* be a mapping. We say that *f* has the approximate endpoint property whenever inf⁡_*x*∈*X*_ *d*(*x*, *fx*) = 0 [1]. Also, the function *g* : ℝ → ℝ is called upper semicontinuous whenever limsup⁡_*n*→*∞*_ *g*(*λ*_*n*_) ≤ *g*(*λ*) for all sequences {*λ*_*n*_}_*n*≥1_ with *λ*_*n*_ → *λ* [2]. In 2010, Amini-Harandi defined the concept of approximate endpoint property for multifunctions and proved the following result (see [1]).

**Theorem**

Let *ψ* : [0, *∞*)→[0, *∞*) be an upper semicontinuous function such that *ψ*(*t*) < *t* and liminf⁡_*t*→*∞*_(*t* − *ψ*(*t*)) > 0 for all *t* > 0, (*X*, *d*) a complete metric space, and *T* : *X* → *CB*(*X*) a multifunction satisfing *H*(*Tx*, *Ty*) ≤ *ψ*(*d*(*x*, *y*)) for all *x*, *y* ∈ *X*. Then *T* has a unique endpoint if and only if *T* has the approximate endpoint property.

Then Moradi and Khojasteh introduced the concept of generalized weak contractive multifunctions and improved Theorem 1 by providing the following result [3].

**Theorem**

Let *ψ* : [0, *∞*)→[0, *∞*) be an upper semicontinuous function such that *ψ*(*t*) < *t* and liminf⁡_*t*→*∞*_(*t* − *ψ*(*t*)) > 0 for all *t* > 0, (*X*, *d*) a complete metric space, and *T* : *X* → *CB*(*X*) a generalized weak contractive multifunction; that is, *T* satisfies *H*(*Tx*, *Ty*) ≤ *ψ*(*N*(*x*, *y*)) for all *x*, *y* ∈ *X*, where *N*(*x*, *y*) = max⁡{*d*(*x*, *y*), *d*(*x*, *Tx*), *d*(*y*, *Ty*), (*d*(*x*, *Ty*) + *d*(*y*, *Tx*))/2}. Then *T* has a unique endpoint if and only if *T* has the approximate endpoint property.

In this paper, we introduce *β*-generalized weak contractive multifunctions, and by adding some conditions to assumptions of the results, we give some results about endpoints of *β*-generalized weak contractive multifunctions. In 2012, the technique of *α*-*ψ*-contractive mappings was introduced by Samet et al. [4]. Later, some authors used it for some subjects in fixed point theory (see for example [5–8]) or generalized it by using the method of *β*-*ψ*-contractive multifunctions (see e.g., [9–12]).

Let (*X*, *d*) be a metric space and *β* : 2^*X*^ × 2^*X*^ → [0, *∞*) a mapping. A multifunction *T* : *X* → 2^*X*^ is called *β*-generalized weak contraction whenever there exists a nondecreasing, upper, semicontinuous function *ψ* : [0, +*∞*)→[0, +*∞*) such that *ψ*(*t*) < *t* for all *t* > 0 and

(1) $$\begin{array}{c} \beta \left(Tx,Ty\right)H\left(Tx,Ty\right)\leq \psi \left(N\left(x,y\right)\right) \end{array}$$

for all *x*, *y* ∈ *X*. We say that *T* is *β*-admissible whenever *β*(*A*, *B*) ≥ 1 implies that *β*(*Tx*, *Ty*) ≥ 1 for all *x* ∈ *A*, and *y* ∈ *B*, where *A* and *B* are subsets of *X*. We say that *T* has the property (*R*) whenever for each convergent sequence {*x*_*n*_} in *X* with *x*_*n*_ → *x* and *β*(*Tx*_*n*−1_, *Tx*_*n*_) ≥ 1 for all *n* ≥ 1, we have *β*(*Tx*_*n*_, *Tx*) ≥ 1. One can find idea of the property (*R*) for mappings in [13]. We say that *T* has the property (*K*) whenever for each sequence {*x*_*n*_} in *X* with *β*(*Tx*_*n*−1_, *Tx*_*n*_) ≥ 1 for all *n* ≥ 1, there exists a natural number *k* such that *β*(*Tx*_*m*_, *Tx*_*n*_) ≥ 1 for all *m* > *n* ≥ *k*. Finally, we say that *T* has the property (*H*) whenever for each *ɛ* > 0, there exists *z* ∈ *X* such that sup⁡_*a*∈*Tz*_ *d*(*z*, *a*) < *ɛ* implies that for every *x* ∈ *X* there exists *y* ∈ *Tx* such that *H*(*Tx*, *Ty*) = sup⁡_*b*∈*Ty*_ *d*(*y*, *b*). A multifunction *T* : *X* → 2^*X*^ is called lower semicontinuous at *x*_0_ ∈ *X* whenever for each sequence {*x*_*n*_} in *X* with *x*_*n*_ → *x*_0_ and every *y* ∈ *Tx*_0_, there exists a sequence {*y*_*n*_} in *X* with *y*_*n*_ ∈ *Tx*_*n*_ for all *n* ≥ 1 such that *y*_*n*_ → *y* [14].

## 2. Main Results

Now, we are ready to state and prove our main results.

**Theorem**

Let (*X*, *d*) be a complete metric space, *β* : 2^*X*^ × 2^*X*^ → [0, *∞*) a mapping, and *T* : *X* → *CB*(*X*) a *β*-admissible, *β*-generalized weak contractive multifunction which has the properties (*R*), (*K*), and (*H*). Suppose that there exist a subset *A* of *X* and *x*_0_ ∈ *A* such that *β*(*A*, *Tx*_0_) ≥ 1. Then *T* has an endpoint if and only if *T* has the approximate endpoint property.

**Proof**

It is clear that if *T* has an endpoint, then *T* has the approximate endpoint property. Conversely, suppose that *T* has the approximate endpoint property. Choose *A* ⊂ *X* and *x*_0_ ∈ *A* such that *β*(*A*, *Tx*_0_) ≥ 1. Since *T* has the approximate endpoint property, for each *ɛ* > 0, there exists *z* ∈ *X* such that sup⁡_*a*∈*Tz*_ *d*(*z*, *a*) < *ɛ*. Now by using the condition (*H*), choose *x*_1_ ∈ *Tx*_0_ such that *H*(*Tx*_0_, *Tx*_1_) = sup⁡_*a*∈*Tx*_1__ *d*(*x*_1_, *a*). Also, choose *x*_2_ ∈ *Tx*_1_ such that *H*(*Tx*_1_, *Tx*_2_) = sup⁡_*a*∈*Tx*_2__ *d*(*x*_2_, *a*), and by continuing this process, we find a sequence {*x*_*n*_} in *X* such that *x*_*n*_ ∈ *Tx*_*n*−1_ and

(2) $$\begin{array}{c} H\left(Tx_{n-1},Tx_{n}\right)=\underset{a\in Tx_{n}}{\sup}d\left(x_{n},a\right), \end{array}$$

for all *n* ≥ 1. Since *β*(*A*, *Tx*_0_) ≥ 1 and *T* is *β*-admissible, *β*(*Tx*_0_, *Tx*_1_) ≥ 1. By using induction, it is easy to see that *β*(*Tx*_*n*−1_, *Tx*_*n*_) ≥ 1 for all *n* ≥ 1. Thus, we obtain

(3) d(xn,xn+1)≤sup⁡a∈Txnd(xn,a)=H(Txn−1,Txn)≤β(Txn−1,Txn)H(Txn−1,Txn)≤ψ(N(xn−1,xn))

for all *n* ≥ 1. If *N*(*x*_*n*−1_, *x*_*n*_) = *d*(*x*_*n*−1_, *x*_*n*_), then

(4) $$\begin{array}{c} d\left(x_{n},x_{n+1}\right)\leq \psi \left(d\left(x_{n-1},x_{n}\right)\right). \end{array}$$

If *N*(*x*_*n*−1_, *x*_*n*_) = *d*(*x*_*n*−1_, *Tx*_*n*−1_), then

(5) $$\begin{array}{c} d\left(x_{n},x_{n+1}\right)\leq \psi \left(d\left(x_{n-1},Tx_{n-1}\right)\right)\leq \psi \left(d\left(x_{n-1},x_{n}\right)\right). \end{array}$$

If *N*(*x*_*n*−1_, *x*_*n*_) = *d*(*x*_*n*_, *Tx*_*n*_), then

(6) $$\begin{array}{c} d\left(x_{n},x_{n+1}\right)\leq \psi \left(d\left(x_{n},Tx_{n}\right)\right)\leq \psi \left(d\left(x_{n},x_{n+1}\right)\right), \end{array}$$

and so *d*(*x*_*n*_, *x*_*n*+1_) = 0. Thus, *d*(*x*_*n*_, *x*_*n*+1_) ≤ *ψ*(*d*(*x*_*n*−1_, *x*_*n*_)). If

(7) N(xn−1,xn)=d(xn,Txn−1)+d(xn−1,Txn)2=d(xn−1,Txn)2d(xn−1,Txn)2≤d(xn−1,xn+1)2≤d(xn−1,xn)+d(xn,xn+1)2≤max⁡{d(xn−1,xn),d(xn,xn+1)},

then *d*(*x*_*n*_, *x*_*n*+1_) ≤ *ψ*(*d*(*x*_*n*−1_, *x*_*n*_)) (other case implies that *d*(*x*_*n*_, *x*_*n*+1_) = 0). Thus,

(8) $$\begin{array}{c} d\left(x_{n},x_{n+1}\right)\leq \psi \left(d\left(x_{n-1},x_{n}\right)\right) \end{array}$$

for all *n* ≥ 1. We claim that *ψ*(0) = 0. If *ψ*(0) > 0, then *ψ* ^2^(0) ≥ *ψ*(0) > 0 because *ψ* is nondecreasing. On the other hand, since *ψ*(*t*) < *t* for all *t* > 0, we have *ψ* ^2^(0) < *ψ*(0) which is a contradiction. Hence, *ψ*(0) = 0. Let *d*_*n*_ = *d*(*x*_*n*_, *x*_*n*+1_) for all *n*. If there exists a natural number *n*_0_ such that *d*_*n*_0__ = 0, then it is easy to see that *d*_*n*_ = 0 for all *n* ≥ *n*_0_, and so lim⁡_*n*→*∞*_ *d*_*n*_ = 0. Now suppose that *d*_*n*_ ≠ 0 for all *n*. In this case, we have *d*_*n*_ ≤ *ψ*(*d*_*n*−1_) < *d*_*n*−1_ for all *n*. Hence, {*d*_*n*_} is a decreasing sequence, and so there exists *d* ≥ 0 such that lim⁡_*n*→*∞*_ *d*_*n*_ = *d*. If *d* > 0, then *d*_*n*_ > 0 for all *n*, and so *d*_*n*_ ≤ *ψ*(*d*_*n*−1_) < *d*_*n*−1_ for all *n*. Since *ψ* is upper and semicontinuous, we obtain *d* = lim⁡_*n*→*∞*_ *d*_*n*_ ≤ lim⁡_*n*→*∞*_ *ψ*(*d*_*n*−1_) ≤ *ψ*(lim⁡_*n*→*∞*_ *d*_*n*−1_) = *ψ*(*d*) < *d* which is a contradiction. Thus, lim⁡_*n*→*∞*_ *d*_*n*_ = 0. Now, we prove that {*x*_*n*_} is a Cauchy sequence. If {*x*_*n*_} is not a Cauchy sequence, then there exist *ɛ* > 0 and natural numbers *m*_*k*_, *n*_*k*_ such that *m*_*k*_ > *n*_*k*_ ≥ *k* and *d*(*x*_*m*_*k*__, *x*_*n*_*k*__) ≥ *ɛ* for all *k* ≥ 1. Also, we choose *m*_*k*_ as small as possible such that

(9) $$\begin{array}{c} d\left(x_{m_{k}-1},x_{n_{k}}\right)<ɛ. \end{array}$$

Thus, *ɛ* ≤ *d*(*x*_*m*_*k*__, *x*_*n*_*k*__) ≤ *d*(*x*_*m*_*k*__, *x*_*m*_*k*_−1_) + *d*(*x*_*m*_*k*_−1_, *x*_*n*_*k*__) ≤ *d*_*m*_*k*_−1_ + *ɛ* for all *k*. Hence, lim⁡_*k*→*∞*_ *d*(*x*_*m*_*k*__, *x*_*n*_*k*__) = *ɛ*. Since *T* has the property (*K*), we obtain

(∗) d(xmk,xnk)≤d(xmk,xmk+1)+d(xmk+1,xnk+1) +d(xnk+1,xnk)≤dmk+H(Txmk,Txnk)+dnk≤dmk+β(Txmk,Txnk) ×H(Txmk,Txnk)+dnk≤dmk+ψ(N(xmk,xnk))+dnk

for all *k*. Since lim⁡_*k*→*∞*_ *d*(*x*_*m*_*k*__, *x*_*n*_*k*__) = *ɛ*, lim⁡_*k*→*∞*_ *N*(*x*_*m*_*k*__, *x*_*n*_*k*__) = *ɛ*. In fact,

(10) d(xmk,xnk) ≤N(xmk,xnk)  =max⁡{d(xmk,xnk),d(xmk,Txmk),d(xnk,Txnk),       d(xmk,Txnk)+d(xnk,Txmk)2} ≤max⁡{d(xmk,xnk),d(xmk,xmk+1),d(xnk,xnk+1),       d(xmk,xnk+1)+d(xnk,xmk+1)2} ≤max⁡{d(xmk,xnk),d(xmk,xmk+1),d(xnk,xnk+1),       (d(xmk,xnk)+d(xnk,xnk+1)         +d(xnk,xmk)+d(xmk,xmk+1))       ×(2)−1},

and so *ɛ* = lim⁡_*k*→+*∞*_ *d*(*x*_*m*_*k*__, *x*_*n*_*k*__) ≤ lim⁡_*k*→*∞*_ *N*(*x*_*m*_*k*__, *x*_*n*_*k*__) ≤ *ɛ*. Since *ψ* is upper semicontinuous, by using ((∗)) we obtain

(11) ɛ=lim⁡k→∞d(xmk,xnk)≤lim⁡k→∞ψ(N(xmk,xnk))≤ψ(ɛ)<ɛ,

which is a contradiction, and so {*x*_*n*_} is a Cauchy sequence. Choose x* ∈ *X* such that *x*_*n*_ → x*. Now, note that

(12) H({xn},Txn)=max⁡⁡{d(xn,Txn),sup⁡y∈Txnd(xn,y)}=H(Txn−1,Txn)

for all *n*, and so

(13) H({xn},Txn)=H(Txn−1,Txn)≤β(Txn−1,Txn)H(Txn−1,Txn)≤ψ(N(xn−1,xn))≤ψ(d(xn−1,xn))≤d(xn−1,xn)

for all *n*, and so lim⁡_*n*→*∞*_ *H*({*x*_*n*_}, *Tx*_*n*_) = 0. Since *T* has the property (*R*), we obtain

(14) H({x∗},Tx∗)≤d(x∗,xn) +H({xn},Txn)+H(Txn,Tx∗)≤d(x∗,xn)+H({xn},Txn) +β(Txn,Tx∗)H(Txn,Tx∗)≤d(x∗,xn)+H({xn},Txn) +ψ(N(xn,x∗))

for all *n*. If *N*(*x*_*n*_, x*) = *d*(x*, Tx*), then we have

(15) H({x∗},Tx∗)  ≤d(x∗,xn)+H({xn},Txn)+ψ(H({x∗},Tx∗))

for all *n*. This implies that *H*({x*}, Tx*) ≤ *ψ*(*H*({x*}, Tx*)), and so

(16) $$\begin{array}{c} H\left(\left\{x^{*}\right\},Tx^{*}\right)=0. \end{array}$$

If *N*(*x*_*n*_, x*) = *d*(*x*_*n*_, x*) or *N*(*x*_*n*_, x*) ≤ *d*(*x*_*n*_, *x*_*n*+1_), then it is easy to see that *H*({x*}, Tx*) = 0. Thus, x* is an endpoint of *T*.

Next example shows that a *β*-generalized weak contractive multifunction is not necessarily a generalized weak contractive multifunction.

**Example**

Let *X* = ℝ. Define *T* : *X* → *CB*(*X*) by *Tx* = [*x*, *x* + 2] for all *x* ∈ *X*. Suppose that *ψ* : [0, +*∞*)→[0, +*∞*) is an arbitrary upper semicontinuous function such that *ψ*(*t*) < *t* for all *t* > 0. If *x* = 0 and *y* = 2, then *H*(*Tx*, *Ty*) = *H*([0,2], [2,4]) = 2 and *N*(*x*, *y*) = 2. Hence,

(17) $$\begin{array}{c} H\left(Tx,Ty\right)=2≩\psi \left(2\right)=\psi \left(N\left(x,y\right)\right). \end{array}$$

Thus, *T* is not a generalized weak contractive multifunction. Now, suppose that *ψ*(*t*) = *t*/2 for all *t* ≥ 0 and define *β* : 2^*X*^ × 2^*X*^ → [0, *∞*) by *β*(*A*, *B*) = 1/2 for all subsets *A* and *B* of *X*. Then, we have

(18) β(Tx,Ty)H(Tx,Ty)=12d(x,y)=ψ(d(x,y))=ψ(N(x,y))

for all *x*, *y* ∈ ℝ. Thus, *T* is a *β*-generalized weak contractive multifunction.

Next example shows that there are multifunctions which satisfy the conditions of Theorem 3, while they are not generalized weak contractive multifunctions.

**Example**

Let *X* = [0, 9/2] and let *d*(*x*, *y*) = |*x* − *y*|. Define *T* : *X* → *CB*(*X*) by

(19) $$\begin{array}{c} Tx=\{\begin{array}{cc} \left\{\frac{x}{2}\right\} & 0\leq x\leq 1\\ \left\{4x-\frac{3}{2}\right\} & 1<x\leq \frac{3}{2}\\ \left\{0\right\} & \frac{3}{2}<x\leq \frac{9}{2}. \end{array} \end{array}$$

If *x* = 1 and *y* = 3/2, then

(20) H(Tx,Ty)=H({12},{92})=4>3=N(x,y)>ψ(N(x,y)),

where *ψ* : [0, +*∞*)→[0, +*∞*) is an arbitrary upper semicontinuous function such that *ψ*(*t*) < *t* for all *t* > 0. Thus, *T* is not a generalized weak contractive multifunction. Now, we show that *T* satisfies all conditions of Theorem 3. For this aim, define *ψ*(*t*) = *t*/2 and *β*(*A*, *B*) = 1 whenever *A* and *B* are subsets of [0,1] and *β*(*A*, *B*) = 0 otherwise. First suppose that *x* ∉ [0,1] or that *y* ∉ [0,1]. If *x*, *y* ∈ (3/2, 9/2], then *Tx*, *Ty* ⊂ [0,1] and *β*(*Tx*, *Ty*) = 1. But, *H*(*Tx*, *Ty*) = 0, and so *β*(*Tx*, *Ty*)*H*(*Tx*, *Ty*) ≤ *ψ*(*N*(*x*, *y*)). If *x* ∈ (1, 3/2] or *y* ∈ (1, 3/2], then *Tx*⊈[0,1], or *Ty*⊈[0,1] and so *β*(*Tx*, *Ty*) = 0. Hence, *β*(*Tx*, *Ty*)*H*(*Tx*, *Ty*) ≤ *ψ*(*N*(*x*, *y*)). Now, suppose that *x*, *y* ∈ [0,1]. In this case, we have *β*(*Tx*, *Ty*) ≥ 1, *H*(*Tx*, *Ty*) = *H*({*x*/2}, {*y*/2}) = (1/2)*d*(*x*, *y*), and *N*(*x*, *y*) = max⁡{*d*(*x*, *y*), *x*/2, *y*/2, (*d*(*x*, *y*/2) + *d*(*y*, *x*/2))/2}. Thus, *d*(*x*, *y*) ≤ *N*(*x*, *y*), and so

(21) β(Tx,Ty)H(Tx,Ty)=12d(x,y)≤ψ(d(x,y))≤ψ(N(x,y)).

Therefore, *T* is a *β*-generalized weak contractive multifunction. Now, we show that *T* is *β*-admissible. If *β*(*A*, *B*) ≥ 1, then *A*, *B* ⊂ [0,1], and so *Tx* = {*x*/2}∈[0,1] and *Ty* = {*y*/2}∈[0,1] for all *x* ∈ *A* and *y* ∈ *B*. Thus, *β*(*Tx*, *Ty*) ≥ 1 for all *x* ∈ *A* and *y* ∈ *B*. Now, suppose *A* = [0, 1/2] and *x*_0_ = 1/4. Then, *Tx*_0_ = {1/8}∈[0,1] and [0, 1/2]⊂[0,1]. Hence, *β*(*A*, *Tx*_0_) ≥ 1. Now, we show that *T* satisfies the condition (*H*). First note that, for each *ɛ* > 0, there exists *z* ∈ *X* such that sup⁡_*a*∈*Tz*_ *d*(*z*, *a*) < *ɛ*. Now, we show that for each *x* ∈ *X* there exists *y* ∈ *Tx* such that *H*(*Tx*, *Ty*) = sup⁡_*b*∈*Ty*_ *d*(*y*, *b*). If 0 ≤ *x* ≤ 1, then *Tx* = {*x*/2}, *T*(*x*/2) = {*x*/4}, and

(22) H(Tx,T(x2))=H({x2},{x4})=x4=sup⁡b∈T(x/2)d(x2,b).

Since for 1 < *x* ≤ 3/2 we have 5/2 < 4*x* − (3/2) ≤ 9/2, *T*(4*x* − (3/2)) = {0}. Thus,

(23) H(Tx,T(4x−32))=H({4x−32},{0})=4x−32=sup⁡b∈T(4x−3/2)d(4x−32,b).

If 3/2 < *x* ≤ 9/2, then *Tx* = {0} and *T*(0) = {0}. Hence,

(24) H(Tx,T(0))=H({0},{0})=0=sup⁡b∈T(0)d(0,b).

It is easy to check that *T* satisfies the conditions (*R*) and (*K*). Note that, 0 is the endpoint of *T*.

Now, we add an assumption to obtain uniqueness of endpoint. In this way, we introduce a new notion. Let *X* be a set and *β* : 2^*X*^ × 2^*X*^ → [0, *∞*) a map. We say that the set *X* has the property (*G*_*β*_) whenever *β*(*A*, *B*) ≥ 1 for all subsets *A* and *B* of *X* with *A*⊈*B* or *B*⊈*A*.

**Corollary**

Let (*X*, *d*) be a complete metric space, *β* : 2^*X*^ × 2^*X*^ → [0, *∞*) a mapping, and *T* : *X* → *CB*(*X*) a *β*-admissible, *β*-generalized weak contractive multifunction which has the properties (*R*), (*K*), and (*H*). Suppose that there exist a subset *A* of *X* and *x*_0_ ∈ *A* such that *β*(*A*, *Tx*_0_) ≥ 1. If *T* has the approximate endpoint property and *X* has the property (*G*_*β*_), then *T* has a unique endpoint.

**Proof**

By using Theorem 3, *T* has a endpoint. If *T* has two distinct endpoints x* and y*, then *β*(Tx*, Ty*) = *β*({x*}, {y*}) ≥ 1 because *X* has the property (*G*_*β*_). Hence,

(25) d(x∗,y∗)≤H(Tx∗,Ty∗)≤β(Tx∗,Ty∗)H(Tx∗,Ty∗)≤ψ(N(x∗,y∗))<N(x∗,y∗)=d(x∗,y∗),

which is a contradiction. Thus, *T* has a unique endpoint.

In Example 5, *T* has a unique endpoint, while *X* does has not the property (*G*_*β*_). Also, *T* has the property (*R*), while *T* is not lower semicontinuous. To see this, consider the sequence {*x*_*n*_} defined by

(26) $$\begin{array}{c} x_{n}=\{\begin{array}{cc} 1-\frac{1}{n} & n=2k\\ 1+\frac{1}{n} & n=2k-1 \end{array} \end{array}$$

for *k* ≥ 1 and put *y* = 1/2 and *x*_0_ = 1. Then *x*_*n*_ → 1 and *y* ∈ *Tx*_0_ = {1/2}. Let {*y*_*n*_} be an arbitrary sequence in *X* such that *y*_*n*_ ∈ *Tx*_*n*_ for all *n* ≥ 1. Then, *y*_2*k*−1_ ∈ *Tx*_2*k*−1_ and *y*_2*k*_ ∈ *Tx*_2*k*_ for all *k*. But, *y*_2*k*−1_ = 4*x*_2*k*−1_ − (3/2) for sufficiently large *k* and *y*_2*k*_ = *x*_2*k*_/2 for all *k* since *y*_2*k*−1_ → 5/2, *y*_*n*_↛1/2. This implies that *T* is not lower semicontinuous.

**Corollary**

Let (*X*, *d*) be a complete metric space, *β* : 2^*X*^ × 2^*X*^ → [0, *∞*) a mapping, and *T* : *X* → *CB*(*X*) a *β*-admissible multifunction which has the properties (*R*), (*K*), and (*H*). Suppose that *X* has the property (*G*_*β*_), and there exist a subset *A* of X, *x*_0_ ∈ *A* and *k* ∈ [0,1) such that *β*(*A*, *Tx*_0_) ≥ 1 and *β*(*Tx*, *Ty*)*H*(*Tx*, *Ty*) ≤ *kN*(*x*, *y*) for all *x*, *y* ∈ *X*. Then *T* has a unique endpoint if and only if *T* has the approximate endpoint property.

**Proof**

It is sufficient that we define *ψ*(*t*) = *kt* for all *t* ≥ 0. Then, Theorem 3 and Corollary 6 guarantee the result.

It has been proved that lower semicontinuity of the multifunction *T* and the property (*R*) are independent conditions [9]. We can replace lower semicontinuity of the multifunction instead of the property (*R*) to obtain the next result. Its proof is similar to the proof of Theorem 3.

**Theorem**

Let (*X*, *d*) be a complete metric space, *β* : 2^*X*^ × 2^*X*^ → [0, *∞*) a mapping, and *T* : *X* → *CB*(*X*) a lower semicontinuous, *β*-admissible, *β*-generalized weak contractive multifunction which has the properties (*K*) and (*H*). Suppose that there exist a subset *A* of *X* and *x*_0_ ∈ *A* such that *β*(*A*, *Tx*_0_) ≥ 1. Then *T* has the approximate endpoint property if and only if *T* has an endpoint.

**Corollary**

Let (*X*, *d*) be a complete metric space, *β* : 2^*X*^ × 2^*X*^ → [0, *∞*) a mapping, and *T* : *X* → *CB*(*X*) a lower semicontinuous, *β*-admissible, *β*-generalized weak contractive multifunction which has the properties (*K*) and (*H*). Suppose that there exist a subset *A* of *X* and *x*_0_ ∈ *A* such that *β*(*A*, *Tx*_0_) ≥ 1. If *T* has the approximate endpoint property and *X* has the property (*G*_*β*_), then *T* has a unique endpoint.

**Corollary**

Let (*X*, *d*) be a complete metric space, *β* : 2^*X*^ × 2^*X*^ → [0, *∞*) a mapping, and *T* : *X* → *CB*(*X*) a *β*-admissible multifunction which has the properties (*R*), (*K*), and (*H*). Suppose that *X* has the property (*G*_*β*_), and there exist a subset *A* of *X*, *x*_0_ ∈ *A* and *k* ∈ [0,1) such that *β*(*A*, *Tx*_0_) ≥ 1 and *β*(*Tx*, *Ty*)*H*(*Tx*, *Ty*) ≤ *kN*(*x*, *y*) for all *x*, *y* ∈ *X*. If *T* has the approximate endpoint property, then Fix⁡(*T*) = End(*T*) = {*x*}.

**Proof**

If we put *ψ*(*t*) = *kt*, then, by using Theorem 2.10 in [9], *T* has a fixed point. Since *T* has the approximate endpoint property, by using Corollary 7, *T* has a unique endpoint such *x*. Let *y* ∈ Fix⁡(*T*). If *Tx* = *Ty*, then *y* = *x*. If *Tx* ≠ *Ty*, then *β*(*Tx*, *Ty*) ≥ 1 because *X* has the property (*G*_*β*_). Also, we have

(27) d(x,y)≤H({x},Ty)=H(Tx,Ty)≤β(Tx,Ty)H(Tx,Ty)≤kN(x,y).

But, *N*(*x*, *y*) = max⁡{*d*(*x*, *y*), *d*(*x*, *Tx*), *d*(*y*, *Ty*), (*d*(*x*, *Ty*) + *d*(*y*, *Tx*))/2} = *d*(*x*, *y*). Thus, *d*(*x*, *y*) = 0, and so Fix⁡(*T*) = End(*T*) = {*x*}.

Next corollary shows us the role of a point in the existence of endpoints.

**Corollary**

Let (*X*, *d*) be a complete metric space, x* ∈ *X* a fixed element, and *T* : *X* → *CB*(*X*) a multifunction such that *T* has the property (*H*) and x* ∈ *Tx*∩*Ty* for all subsets *A* and *B* of *X* with x* ∈ *A*∩*B* and all *x* ∈ *A* and *y* ∈ *B*. Assume that *H*(*Tx*, *Ty*) ≤ *ψ*(*N*(*x*, *y*)) for all *x*, *y* ∈ *X* with x* ∈ *Tx*∩*Ty*, where *ψ* : [0, +*∞*)→[0, +*∞*) is a nondecreasing upper semicontinuous function such that *ψ*(*t*) < *t* for all *t* > 0. Suppose that there exist a subset *A*_0_ of *X* and *x*_0_ ∈ *A*_0_ such that x* ∈ *A*_0_∩*Tx*_0_. Assume that for each convergent sequence {*x*_*n*_} in *X* with *x*_*n*_ → *x* and x* ∈ *Tx*_*n*−1_∩*Tx*_*n*_, for all *n* ≥ 1, one has x* ∈ *Tx*_*n*_∩*Tx*. Also, for each sequence {*x*_*n*_} in *X* with x* ∈ *Tx*_*n*−1_∩*Tx*_*n*_ for all *n* ≥ 1, there exists a natural number *k* such that x* ∈ *Tx*_*m*_∩*Tx*_*n*_ for all *m* > *n* ≥ *k*. Then *T* has an endpoint if and only if *T* has the approximate endpoint property.

**Proof**

It is sufficient we define *β* : 2^*X*^ × 2^*X*^ → [0, *∞*) by *β*(*A*, *B*) = 1 whenever x* ∈ *A*∩*B* and *β*(*A*, *B*) = 0 otherwise, and then we use Theorem 3.

**Corollary**

Let (*X*, *d*) be a complete metric space, x* ∈ *X* a fixed element and *T* : *X* → *CB*(*X*) a lower semicontinuous multifunction such that *T* has the property (*H*) and x* ∈ *Tx*∩*Ty* for all subsets *A* and *B* of *X* with x* ∈ *A*∩*B* and all *x* ∈ *A* and *y* ∈ *B*. Assume that

(28) $$\begin{array}{c} H\left(Tx,Ty\right)\leq \psi \left(N\left(x,y\right)\right) \end{array}$$

for all *x*, *y* ∈ *X* with x* ∈ *Tx*∩*Ty*, where *ψ* : [0, +*∞*)→[0, +*∞*) is a nondecreasing upper semicontinuous function such that *ψ*(*t*) < *t* for all *t* > 0. Suppose that there exist a subset *A*_0_ of *X* and *x*_0_ ∈ *A*_0_ such that x* ∈ *A*_0_∩*Tx*_0_. Assume that for each convergent sequence {*x*_*n*_} in *X* with *x*_*n*_ → *x* and x* ∈ *Tx*_*n*−1_∩*Tx*_*n*_ for all *n* ≥ 1, we have x* ∈ *Tx*_*n*_∩*Tx*. Then *T* has an endpoint if and only if *T* has the approximate endpoint property.

**Proof**

It is sufficient to define *β* : 2^*X*^ × 2^*X*^ → [0, *∞*) by *β*(*A*, *B*) = 1 whenever x* ∈ *A*∩*B* and *β*(*A*, *B*) = 0 otherwise, and then we use Theorem 8.

Let (*X*, *d*, ≤) be an ordered metric space. Define the order ⪯ on arbitrary subsets *A* and *B* of *X* by *A*⪯*B* if and only if for each *a* ∈ *A* there exists *b* ∈ *B* such that *a* ≤ *b*. It is easy to check that (*CB*(*X*), ⪯) is a partially ordered set.

**Theorem**

Let (*X*, *d*, ≤) be a complete ordered metric space and *T* a closed and bounded valued multifunction on *X* such that *T* has the property (*H*) and *Tx*⪯*Ty* for all subsets *A* and *B* of *X* with *A*⪯*B* and all *x* ∈ *A* and *y* ∈ *B*. Assume that *H*(*Tx*, *Ty*) ≤ *ψ*(*N*(*x*, *y*)) for all *x*, *y* ∈ *X* with *Tx*⪯*Ty*, where *ψ* : [0, +*∞*)→[0, +*∞*) is a nondecreasing upper semicontinuous function such that *ψ*(*t*) < *t* for all *t* > 0. Suppose that there exist a subset *A*_0_ of *X* and *x*_0_ ∈ *A*_0_ such that *A*_0_⪯*Tx*_0_. Assume that for each convergent sequence {*x*_*n*_} in *X* with *x*_*n*_ → *x* and *Tx*_*n*−1_⪯*Tx*_*n*_, for all *n* ≥ 1, one has *Tx*_*n*_⪯*Tx*. Also, for each sequence {*x*_*n*_} in *X* with *Tx*_*n*−1_⪯*Tx*_*n*_ for all *n* ≥ 1, there exists a natural number *k* such that *Tx*_*m*_⪯*Tx*_*n*_ for all *m* > *n* ≥ *k*. Then *T* has an endpoint if and only if *T* has the approximate endpoint property.

**Proof**

Define *β*(*A*, *B*) = 1 whenever *A*⪯*B* and *β*(*A*, *B*) = 0 otherwise, and then we use Theorem 3.

**Corollary**

Let (*X*, *d*, ≤) be a complete ordered metric space and *T* a closed and bounded valued multifunction on *X* such that *T* has the property (*H*) and *Tx*⪯*Ty* for all subsets *A* and *B* of *X* with *A*⪯*B*, all *x* ∈ *A*, and *y* ∈ *B*. Assume that *H*(*Tx*, *Ty*) ≤ *ψ*(*N*(*x*, *y*)) for all *x*, *y* ∈ *X* with *Tx*⪯*Ty*, where *ψ* : [0, +*∞*)→[0, +*∞*) is a nondecreasing upper semicontinuous function such that *ψ*(*t*) < *t* for all *t* > 0. Suppose that there exist a subset *A*_0_ of *X* and *x*_0_ ∈ *A*_0_ such that *A*_0_⪯*Tx*_0_. Assume that for each convergent sequence {*x*_*n*_} in *X* with *x*_*n*_ → *x* and *Tx*_*n*−1_⪯*Tx*_*n*_, for all *n* ≥ 1, one has *Tx*_*n*_⪯*Tx*. Also, for each sequence {*x*_*n*_} in *X* with *Tx*_*n*−1_⪯*Tx*_*n*_ for all *n* ≥ 1, there exists a natural number *k* such that *Tx*_*m*_⪯*Tx*_*n*_ for all *m* > *n* ≥ *k*. If *T* has the approximate endpoint property and *A*⪯*B* for all subsets *A* and *B* of *X* with *A*⊈*B* or *B*⊈*A*, then *T* has a unique endpoint.

**Proof**

Define *β*(*A*, *B*) = 1 whenever *A*⪯*B* and *β*(*A*, *B*) = 0 otherwise, and then we use Corollary 6.

Let (*X*, *d*) be a metric space and *T* : *X* → 2^*X*^ a multifunction. We say that *T* is an *HS*-multifunction whenever for each *x* ∈ *X* there exists *y* ∈ *Tx* such that *H*(*Tx*, *Ty*) = sup⁡_*b*∈*T*y_ *d*(*y*, *b*). It is obvious that each *HS*-multifunction is an multifunction which has the property (*H*). Thus, one can conclude similar results to above ones for *HS*-multifunctions. Here, we provide some ones. Although by considering *HS*-multifunction we restrict ourselves, we obtain strange results with respect to above ones. One can prove the following by reading exactly the proofs of similar above results.

**Theorem**

Let (*X*, *d*) be a complete metric space, *β* : 2^*X*^ × 2^*X*^ → [0, *∞*) a mapping, and *T* : *X* → *CB*(*X*) a *β*-admissible, *β*-generalized weak contractive *HS*-multifunction which has the properties (*R*) and (*K*). Suppose that there exist a subset *A* of *X* and *x*_0_ ∈ *A* such that *β*(*A*, *Tx*_0_) ≥ 1. Then *T* has an endpoint, and so *T* has the approximate endpoint property.

**Theorem**

Let (*X*, *d*) be a complete metric space, *β* : 2^*X*^ × 2^*X*^ → [0, *∞*) a mapping, and *T* : *X* → *CB*(*X*) a lower semicontinuous, *β*-admissible, and *β*-generalized weak contractive *HS*-multifunction which has the property (*K*). Suppose that there exist a subset *A* of *X* and *x*_0_ ∈ *A* such that *β*(*A*, *Tx*_0_) ≥ 1. Then *T* has an endpoint, and so *T* has the approximate endpoint property.

The next result is a consequence of Theorem 15.

**Corollary**

Let (*X*, *d*) be a complete metric space, x* ∈ *X* a fixed element, and *T* : *X* → *CB*(*X*) an *HS*-multifunction such that x* ∈ *Tx*∩*Ty* for all subsets *A* and *B* of *X* with x* ∈ *A*∩*B*, all *x* ∈ *A*, and *y* ∈ *B*. Assume that *H*(*Tx*, *Ty*) ≤ *ψ*(*N*(*x*, *y*)) for all *x*, *y* ∈ *X* with x* ∈ *Tx*∩*Ty*, where *ψ* : [0, +*∞*)→[0, +*∞*) is a nondecreasing upper semicontinuous function such that *ψ*(*t*) < *t* for all *t* > 0. Suppose that there exist a subset *A*_0_ of *X* and *x*_0_ ∈ *A*_0_ such that x* ∈ *A*_0_∩*Tx*_0_. Assume that for each convergent sequence {*x*_*n*_} in *X* with *x*_*n*_ → *x* and x* ∈ *Tx*_*n*−1_∩*Tx*_*n*_ for all *n* ≥ 1 one has x* ∈ *Tx*_*n*_∩*Tx*. Also, for each sequence {*x*_*n*_} in *X* with x* ∈ *Tx*_*n*−1_∩*Tx*_*n*_ for all *n* ≥ 1, there exists a natural number *k* such that x* ∈ *Tx*_*m*_∩*Tx*_*n*_ for all *m* > *n* ≥ *k*. Then *T* has an endpoint, and so *T* has the approximate endpoint property.

The next result is a consequence of Theorem 16.

**Corollary**

Let (*X*, *d*, ≤) be a complete ordered metric space and *T* a closed and bounded valued lower semicontinuous *HS*-multifunction on *X* such that *Tx*⪯*Ty* for all subsets *A* and *B* of *X* with *A*⪯*B*, all *x* ∈ *A*, and *y* ∈ *B*. Assume that *H*(*Tx*, *Ty*) ≤ *ψ*(*N*(*x*, *y*)) for all *x*, *y* ∈ *X* with *Tx*⪯*Ty*, where *ψ* : [0, +*∞*)→[0, +*∞*) is a nondecreasing upper semicontinuous function such that *ψ*(*t*) < *t* for all *t* > 0. Suppose that there exist a subset *A*_0_ of *X* and *x*_0_ ∈ *A*_0_ such that *A*_0_⪯*Tx*_0_. Assume that for each sequence {*x*_*n*_} in *X* with *Tx*_*n*−1_⪯*Tx*_*n*_ for all *n* ≥ 1, there exists a natural number *k* such that *Tx*_*m*_⪯*Tx*_*n*_ for all *m* > *n* ≥ *k*. Then *T* has an endpoint, and so *T* has the approximate endpoint property.
